# Optimization of High-Moisture Meat Analog Production with the Addition of Isolated Mung Bean Protein Using Response Surface Methodology

**DOI:** 10.3390/foods14081323

**Published:** 2025-04-11

**Authors:** Yu Zhang, Bon-Jae Gu, Nam-ki Hwang, Gi-Hyung Ryu

**Affiliations:** 1 Department of Food and Quality Engineering, Nanning University, Nanning 530200, China; zhangyu1472@outlook.com; 2 Food and Feed Extrusion Research Center, Department of Food Science and Technology, Kongju National University, Yesan 32439, Republic of Korea; bon-jae.gu@kongju.ac.kr (B.-J.G.);

**Keywords:** isolated mung bean protein, high-moisture extrusion cooking, meat analog, process variables, response surface methodology

## Abstract

Meat analogs focus on sustainable development, mimicking the physical properties and nutritional components of meat. The main objective of this study was to determine the optimal extrusion process parameters for producing high-moisture meat analogs (HMMAs) by adding 30% isolated mung bean protein (IMBP) using the response surface methodology (RSM). This study evaluated the effects of independent variables (moisture content—50%, 55%, and 60%; screw speed—150, 200, and 250 rpm; and barrel temperature—140, 150, and 160 °C) on the physicochemical and textural properties of the meat analogs during high-moisture extrusion. The results indicated that moisture content had a greater impact compared to barrel temperature and screw speed. Furthermore, the fiber structure increased with rising barrel temperature, while increasing moisture content led to a reduction in fiber structure. The water absorption capacity and nitrogen solubility index were positively correlated with moisture content, whereas the oil absorption capacity, integrity index, chewiness, and cutting strength showed the opposite trend. The study predicted the optimal extrusion process parameters for IMBP-based HMMAs to be a moisture content of 54.21%, screw speed of 185.68 rpm, and barrel temperature of 159.36 °C. Considering practical conditions, the optimal process variables for producing IMBP-based HMMAs in this experiment were adjusted to a moisture content of 54%, screw speed of 186 rpm, and barrel temperature of 159 °C.

## 1. Introduction

Meat analogs are foods made from plant proteins (such as soy protein, pea protein, wheat protein, etc.) that mimic the appearance, texture, and nutritional content of animal meat [1]. The plant-based meat market has grown rapidly in recent years. According to market research reports, the global plant-based meat market size was approximately $4.3 billion in 2021 and is expected to reach $8.5 billion by 2027, with a compound annual growth rate of about 10% [2]. This growth is driven by increasing consumer awareness of healthy eating [3], environmental protection, and animal welfare [4]. The variety of plant-based meat products is becoming increasingly diverse, expanding from initial products like plant-based burgers and sausages to a wider range of items such as plant-based chicken nuggets, fish fillets, and steaks [5].

Technological advancements are a crucial driving force in the development of the plant-based meat industry. Techniques such as extrusion, 3D printing, and molecular biology are improving the texture and flavor of plant-based meat. For example, 3D printing technology is used to create plant-based meat products with complex textures [6], while cell culture technology explores the combination of plant cells with animal cells to produce more realistic “plant-based meat” [7]. However, extrusion technology has always been the most used technique for producing meat analogs. Extrusion cooking is currently considered a technique for manufacturing artificial meat through high temperature, high pressure, and shear forces [8,9]. Proteins undergo thermal and mechanical stress through the heating barrel and shearing screw, leading to the melting, intensive mixing, and structuring of protein molecules. By installing a long cooling die at the end of the extruder, it is assumed that the proteins realign in the flow direction, forming an anisotropic protein network [10,11,12]. In the high-moisture extrusion cooking (HMEC) process, key process variables include moisture content, screw speed, and barrel temperature, which significantly impact the quality and characteristics of the final product. By optimizing these variables, it is possible to produce high-quality plant-based meat products with textures and functional properties that are closer to those of animal meat.

Mung bean protein, as an emerging plant protein source, shows significant advantages in the production of meat substitutes due to its various superior characteristics. Mung bean protein has high nutritional value, is rich in essential amino acids [13], and is a low-fat, low-calorie protein source without cholesterol [14], making it a healthy meat substitute. Additionally, compared to other common plant proteins (such as soy protein and wheat protein), mung bean protein is free from common allergens [15]. Mung bean protein has good water solubility and water absorption [16], making it easier to handle food processing and helping to improve the texture of the product. Seetapan et al. [17] reported that a mixture of isolated mung bean protein (IMBP) and mung bean flour is a promising ingredient for preparing mung bean-based high-moisture meat analogs (HMMAs) with customized texture characteristics. Brishti et al.’s [18] research successfully demonstrated the feasibility of optimizing the production of textured mung bean protein (TMBP) from mung bean protein. TMBP can serve as a meat thickener and is a healthier option compared to animal protein.

Current research mainly focuses on the feasibility of using mung bean protein for producing meat analogs [17,18,19,20], but studies on optimizing the process parameters for high-moisture meat analogs produced by twin-screw extrusion have yet to be reported. Therefore, the aim of this experiment is to investigate the effects of moisture content, screw speed, and barrel temperature on the physicochemical properties (water holding capacity, water absorption capacity, oil absorption capacity, integrity index, nitrogen solubility index) and texture characteristics (texture profile analysis and cutting strength) of high-moisture meat analogs based on isolated mung bean protein. Additionally, the experiment utilizes response surface methodology to optimize the three process variables to obtain the optimal extrusion conditions.

While previous studies have explored the feasibility of mung bean protein in meat analogs [17,18,19,20], this is the first study to systematically optimize the extrusion parameters (moisture, screw speed, and temperature) for HMMAs using response surface methodology (RSM). Unlike previous works focusing only on texture or protein structure, this study provides a comprehensive evaluation of both the physicochemical (water holding capacity, water and oil absorption, integrity index, nitrogen solubility index, etc.) and textural properties (TPA, cutting strength) of IMBP-based HMMAs to achieve the best possible meat-like texture and functionality. To provide scientific insights into the potential of mung bean protein as a high-quality alternative to conventional plant proteins (e.g., soy or wheat protein) in meat analog production. The optimized extrusion conditions could facilitate the commercial production of mung bean-based meat analogs with improved texture and nutritional quality, offering a viable alternative to soy or pea protein-based products.

## 2. Materials and Methods

### 2.1. Materials

The study utilized the following raw materials: isolated mung bean protein (IMBP), isolated soy protein (ISP), wheat gluten (WG), and corn starch (CS). The IMBP was supplied by Harbin Hada Starch Co. (Heilongjiang, China), ISP was sourced from Pingdingshan Tianjing Plant Albumen Co. (Henan, China), WG was obtained from Roquette Freres (Lestrem, France), and CS was provided by Samyang Co. (Ulsan, Republic of Korea). These ingredients were blended at ratios of 30% (IMBP), 20% (ISP), 40% (WG), and 10% (CS).

### 2.2. High-Moisture Extrusion Process

**Figure 1:**
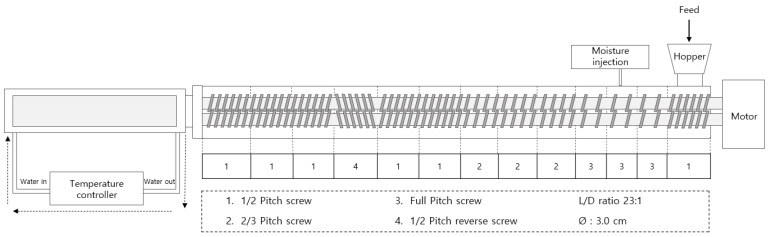
Schematic diagram of high-moisture extrusion system with details of the cooling die and screw configuration.

The high-moisture meat analogs (HMMAs) were produced using isolated mung bean protein (IMBP) in a co-rotating twin-screw extruder (THK31T-5, Incheon Machinery Co., Ltd., Incheon, Republic of Korea) featuring a 30 mm screw diameter with 23:1 length-to-diameter ratio. As shown in Figure 1, the powdered raw material was continuously fed at 100 g/min and transported through the barrel to the long cooling die, which promoted protein alignment and fiber formation to produce structured meat analogs. Extrusion parameters, including moisture content, screw speed, and barrel temperature, were controlled according to the experimental design (Table 1). Immediately after extrusion, the HMMA strands were cut into uniform 1 cm^3^ cubes for analysis. For characterization, samples were divided with one portion vacuum-sealed and stored at 4 °C for integrity index, texture profile analysis (TPA), and cutting strength measurements, while another portion was freeze-dried for water-holding capacity (WHC) determination. The freeze-dried samples were subsequently ground into fine powder, sieved to ensure uniformity, and used for assessing water absorption capacity (WAC), oil absorption capacity (OAC), and nitrogen solubility index (NSI).

### 2.3. Water Holding Capacity (WHC)

The WHC of IMBP-based HMMAs was determined using a modified version of Lin et al.’s method [21]. Freeze-dried samples (3 g dry basis) were hydrated in 100 mL distilled water at 50 °C (SHWB-45 water bath, Lab House, Jakarta Selatan, Indonesia) for 16 h. After hydration, the samples were drained through a 20-mesh sieve for 15 min to remove unabsorbed water. WHC was calculated according to Equation (1), with measurements performed in triplicate.

WHC (%) = (D_2_ − D_1_)/D_1_ × 100 (1)

where D_1_ is the weight of the dried sample, and D_2_ is the weight of rehydrated sample.

### 2.4. Water Absorption Capacity (WAC) and Oil Absorption Capacity (OAC)

The WAC and OAC measurements were conducted following the modified methodology described by Samard and Ryu [22]. Precisely 0.5 g of the ground sample was homogenized with 5 mL of distilled water using a vortex mixer (Model SI-0246A, Vortex-Genie-2, Scientific Industries Inc., New York, NY, USA) for 1 min. The mixture was then incubated in a thermostatic shaker (Model SI-300R, Jelotech, Gangneung, Republic of Korea) at 30 °C for 30 min with continuous agitation, followed by centrifugation at 3000 rpm for 30 min. The supernatant was carefully decanted, and the sediment was retained for analysis. The same procedure was replicated using refined soybean oil instead of distilled water to determine the OAC. The WAC and OAC values of the sediment were determined using Equations (2) and (3), respectively. The densities of distilled water and soybean oil used in the calculations were 1.00 g/cm^3^ and 0.90 g/cm^3^, respectively. All measurements were performed in triplicate, and mean values were reported.

WAC (%) = Weight of sediment/Weight of dry solid(2)

OAC (%) = Weight of sediment/Weight of dry solid(3)

### 2.5. Integrity Index and Nitrogen Solubility Index (NSI)

The structural stability of HMMAs was evaluated through integrity index determination following Gu and Ryu [23]. Freeze-dried samples (4 g) were subjected to 121 °C autoclaving for 15 min in 100 mL distilled water. After rapid cooling, samples were homogenized (IKA-T10B) in fresh distilled water and filtered through a 20-mesh sieve. The retained residue was rinsed and oven-dried at 105 °C. The integrity index was calculated as Equation (4). The NSI was determined using a modified method [24]. For soluble nitrogen, 0.1 g samples were extracted in 0.5% KOH (30 °C, 120 rpm, 20 min) followed by centrifugation (3000 rpm, 30 min). Total nitrogen content was obtained after acid hydrolysis (6 N HCl, 100 °C, 24 h). Both fractions were analyzed using the anthrone method [25], with NSI calculated as Equation (5):

Integrity index (%) = Dry residue weight/Sample weight × 100(4)

NSI (%) = Soluble nitrogen content/Total nitrogen content × 100(5)

### 2.6. Texture Profile Analysis (TPA) and Cuttings Strength

The textural properties of IMBP-based HMMAs were evaluated using a texture analyzer (Compac-100Ⅱ, Sun Sci Corporation, Tokyo, Japan) following the TPA method. A cylindrical probe (25 mm in diameter) was employed to compress samples to 75% deformation of their original height (10 mm) at a constant speed of 100 mm/min under a maximum load of 10 kg for determining springiness, cohesiveness, and chewiness. For cutting strength measurement, a rectangular probe (7.5 mm × 38.3 mm) was used to assess samples in both vertical and parallel directions under a maximum load of 2 kg. For each test condition, ten randomly selected cubic samples (10 mm × 10 mm × 10 mm) were analyzed. After excluding the maximum and minimum values, the mean of the remaining eight measurements was calculated and reported as the result. All measurements were performed at ambient temperature (25 ± 1 °C) with a waiting time of 5 s between two consecutive compressions during TPA testing.

### 2.7. Design and Statistics Analysis

The experimental design adopted the response surface methodology (RSM, Box–Behnken). Design-Expert software version 8 was used to generate experimental design, perform statistical analysis, and develop the regression model. Three independent variables were selected: moisture content (X_1_), screw speed (X_2_), and barrel temperature (X_3_), each with three levels. According to the Box–Behnken configuration, a total of 15 different combinations were randomly chosen (including three replicate center points). The response functions (Y) measured were the water-holding capacity, integrity index, and chewiness of an HMMA with added IRP. These values were related to the coded variables (Xi, i = 1, 2, and 3) through the following second-order polynomial equation:

Y = b_0_ + b_1_X_1_ + b_2_X_2_ + b_3_X_3_ + b_12_X_1_X_2_ + b_13_X_1_X_3_ + b_23_X_2_X_3_ + b_11_X_12_ + b_22_X_22_ + b_33_X_32_

An analysis of variance (ANOVA) table was generated, and the effects and regression coefficients of each linear, quadratic, and interaction term were determined. The significance of all terms in the polynomial was statistically determined by calculating the F-value at a probability (P) of 0.01 or 0.05. Contour plots were then generated based on the regression model using the regression coefficients for statistical calculations.

Data were analyzed using IBM SPSS software version 22.0 (IBM, Armonk, NY, USA). Duncan’s multiple range test was used to compare the means. Differences between treatments were considered significant when *p* < 0.05.

## 3. Results

### 3.1. Fiber Structure

**Figure 2:**
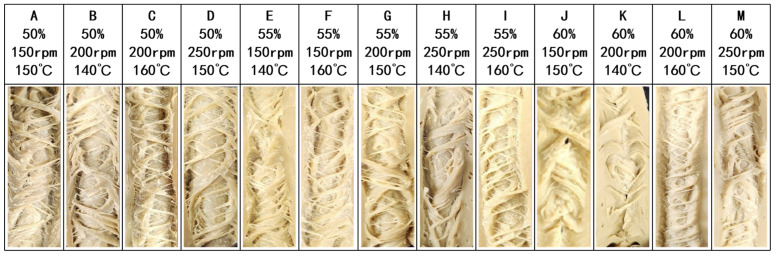
The fibrous structure of isolated mung bean protein-based high-moisture meat analogs obtained under different moisture contents (50, 55, and 60%), barrel temperatures (140, 150, and 160 °C), and screw speeds (150, 200, and 250 rpm) in response surface methodology experiments.

Figure 2 illustrates the fibrous structure of high-moisture meat analogs (HMMAs) based on isolated mung bean protein (IMBP-based) under different process variables. As moisture content and screw speed increase, the fibrous structure decreases. This occurs because higher moisture content results in water filling the gaps between denatured protein structures, leading to a sponge-like texture and reduced fibrous structure due to weakened non-covalent interactions and disulfide bonds among proteins [26]. In high-moisture environments, water molecules penetrate the gaps between proteins, resulting in the formation of competitive hydrogen bonds and the breakdown of the original hydrogen bond network. Additionally, this process increases the distance between protein molecules and hinders the reorganization of disulfide bonds. Barrel temperature significantly influences the fibrous structure formation of plant-based meat substitutes [27]. Increasing barrel temperature enhances the fibrous structure of IMBP-based HMMAs. This is attributed to increased protein molecule separation at higher temperatures, creating additional cross-linking sites that promote fibrous structure formation [28]. Temperature rise promotes the unfolding of protein molecules, exposing the internal hydrophobic core and free-SH groups. These newly exposed active sites aggregate through hydrophobic interactions and oxidize to form disulfide bonds. Under continuous intermolecular interactions, a dense, three-dimensional network structure is ultimately constructed. This structure not only enhances the fibrous texture of the product but also significantly improves water-holding capacity through a mesh-based water-locking mechanism. This explains the dual effect observed in the experiment where high temperature (159 °C) and moderate moisture (54%) synergistically maintain the fibrous structure while optimizing water retention. Additionally, Lin et al. [21] also reported an increase in fibrous structure at barrel temperatures of 138 °C, 149 °C, and 160 °C.

### 3.2. Water Holding Capacity (WHC)

The aim of this experiment was to determine how much water meat analogs could absorb and retain after hydration. WHC is influenced by protein hydrophilic groups, protein primary structure, the presence of carbohydrates, and capillary action [29]. The WHC of IMBP-based HMMAs was significantly influenced by extrusion parameters (Table 2). Maximum WHC (251.66%) was achieved at 50% moisture content, 200 rpm screw speed, and 140 °C barrel temperature. Contrary to the fibrous structure formation trends, WHC exhibited a positive correlation with moisture content (samples A/J, B/K, C/L, and D/M comparisons), as higher hydration levels facilitated greater water-protein interactions through exposed hydrophilic groups and capillary action [29]. Notably, WHC increased with barrel temperature (samples B/C, E/F, and K/L comparisons). At fixed screw speeds, WHC peaked at 60% moisture content and 160 °C, while at constant temperatures, maximum WHC occurred at 60% moisture and 200 rpm. This thermal enhancement effect can be attributed to protein structural reorganization—elevated temperatures promote protein denaturation and subsequent realignment, forming a more interconnected network with improved water entrapment capabilities [30]. Specifically, the “tightening” effect refers to thermally induced protein-protein interactions (including disulfide bridging and hydrophobic associations) that create a denser yet more water-retentive matrix, as opposed to the more porous structure formed at lower temperatures. These findings align with Brishti et al. [18], who similarly observed WHC improvement (at 45% moisture) with temperature increases from 100–150 °C.

### 3.3. Water Absorption Capacity (WAC) and Oil Absorption Capacity (OAC)

The effects of different process variables on the water absorption and oil absorption capacities of high-moisture meat analogs based on isolated mung bean protein are detailed in Table 2. The WAC of HMMAs ranges from 298.51% to 425.66%. WAC depends on the number of polar sites on proteins interacting with water [11] and is influenced by protein primary structure, hydrophilicity, and the presence of carbohydrates [29]. During extrusion molding, under high temperature and pressure conditions leading to protein denaturation, exposure of surface hydrophilic groups increases, thereby enhancing WAC [11]. As moisture content, screw speed, and barrel temperature increase, WAC also increases. Higher moisture content reduces the viscosity of the mixture inside the barrel, leading to incomplete protein denaturation, decreased protein-protein interactions, and cross-linking. The screw speed determines the extent of mixture denaturation, influencing the quality of the meat substitute [31]. Increased screw speed reduces the residence time of the mixture, increasing the binding of hydrophilic residues of inadequately denatured protein structures with water [21,32].

The OAC of HMMAs ranges from 130.61% to 193.56%. OAC is a critical food characteristic that contributes to improving the satiety, flavor, and texture of foods. This capacity is influenced by the presence of hydrophobic residues on the protein surface [29]. The impact of screw speed and barrel temperature on OAC is minimal compared to moisture content. However, OAC increases when moisture content reaches 55–56% and then decreases thereafter. During extrusion molding, an increase in moisture content to 55–56% causes denaturation of mung bean protein, exposing hydrophobic residues and thereby enhancing OAC [20]. The subsequent decrease in OAC occurs because moisture content exceeding 55–56% reduces the viscosity of the mixture, decreases friction, and lowers protein denaturation and exposure of hydrophobic residues [33]. These findings suggest that for optimal oil absorption in sauces or soups, a moisture content of 55–56%, screw speed of 200–225 rpm, and barrel temperature of 150 °C, or a moisture content of 55–56%, screw speed of 200 rpm, and barrel temperature of 150–160 °C are favorable conditions for meat analogs.

### 3.4. Integrity Index and Nitrogen Solubility Index (NSI)

IMBP-based HMMAs’ integrity index ranges from 62.67% to 96.47%. As moisture content increases, the integrity index decreases, while increasing screw speed increases it. Increased moisture content reduces the viscosity and temperature of the mixture, leading to incomplete protein denaturation and reduced protein-protein interactions and cross-linking, resulting in a softer meat substitute [34]. This is consistent with Asgar et al.’s [35] study on hemp seed protein-based meat substitutes, where increased moisture content decreases the integrity index. On the other hand, increased screw speed increases protein dispersion and cross-linking, producing a firmer meat substitute [32].

IMBP-based HMMAs’ NSI ranges from 26.60% to 46.13%. Increased moisture content and barrel temperature increase the NSI. Increased moisture content reduces the thermal energy and shear forces acting on the melt, decreasing protein structure unfolding and new protein binding [36]. Increased barrel temperature changes the protein structure, exposing more hydrophobic regions, and strong protein-water interactions lead to a decrease in the NSI [37]. In contrast, the screw speed’s impact on the IMBP-based HMMA’s NSI is not significantly clear.

### 3.5. Texture Profile Analysis and Cutting Strength

The objective assessment of meat analogs’ resistance to physical forces is evaluated through TPA and cutting strength values. Springiness represents the proportion of sample recovery after applying deformation, while cohesiveness indicates the strength of the formed network [38]. Table 3 shows the effects of different process variables on TPA and cutting strength of HMMAs based on IMBP. The range of springiness and cohesiveness for IMBP-based HMMAs is 92.56% to 96.40% and 81.46% to 87.11%, respectively. Springiness and cohesiveness are minimally affected by moisture content, barrel temperature, and screw speed. This is like findings by Maung et al. [28] on the manufacturing of high-moisture meat substitutes, indicating no significant differences in springiness and cohesiveness. The range of chewiness for IMBP-based HMMAs is 2301.10 to 7089.33 g. Chewiness decreases with increasing moisture content. Chen et al. [39] reported that increased moisture content reduces protein aggregation and the re-binding of protein molecules. Therefore, increased moisture content leads to lower viscosity and elasticity within the barrel, making the texture of high-moisture meat substitutes softer [40].

The vertical direction cutting strength ranges from 686.79 to 1595.01 g/cm^2^, and the parallel direction cutting strength ranges from 645 to 1027.58 g/cm^2^ for IMBP-based HMMAs. As moisture content and screw speed increase, cutting strength shows a decreasing trend. This is because increased moisture content results in incomplete denaturation of the mixture, reducing protein-protein interactions and cross-linking [32]. Studies by Lin et al., Palanisamy et al., and Zahari et al. [21,32,41] corroborated the findings of reduced chewiness and cutting strength in HMMAs as observed in this study.

### 3.6. Optimization of Process Variables

**Figure 3:**
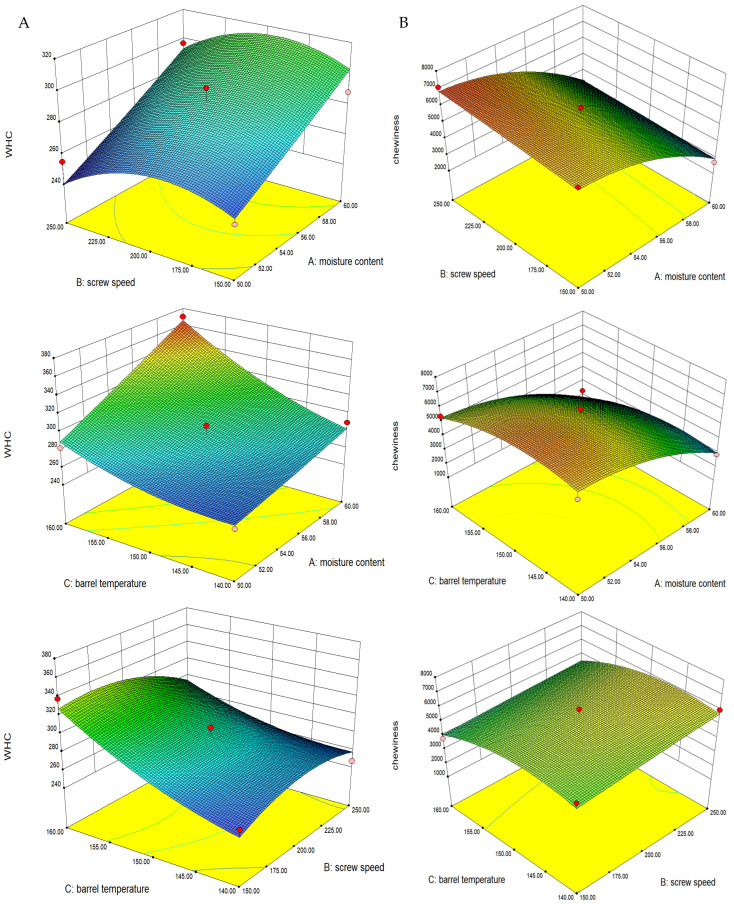
Contour plots of the response surface for water holding capacity (**A**) and chewiness (**B**) of isolated mung bean protein-based high-moisture meat analogs, influenced by moisture contents (50, 55, and 60%), barrel temperatures (140, 150, and 160 °C), and screw speeds (150, 200, and 250 rpm).

Texture and mouthfeel are critical characteristics of meat substitutes, as consumers expect these alternatives to mimic the feel of real meat. For meat analog products, WHC and chewiness are important factors determining their quality. The effects of different process variables (moisture content, barrel temperature, and screw speed) on WHC and chewiness are expressed in Table 4 using quadratic polynomial coefficients. For clarity, the response contour plots for WHC and chewiness are shown in Figure 3A and Figure 3B respectively. The final equations calculated for WHC and chewiness using coded factors are as follows:

WHC = 287.53 + 25.94 * X1 − 5.53 * X2 + 29.35 * X3 + 0.65 * X1 * X2 + 12.58 * X1 * X3 − 10.68 * X2 * X3 + 0.78 * X12 − 15.97 * X22 + 10.19 * X32

Chewiness = 5649.38 − 1659.40 * X1 + 301.35 * X2 − 510.80 * X3−64.12 * X1 * X2 − 93.92 * X1 * X3 − 70.95 * X2 * X3 − 797.30 * X12 − 19.69 *X22 − 794.67 * X32

From Table 4, it is evident that the model is suitable for WHC (*p* < 0.05, lack of fit > 0.05) and chewiness (*p* < 0.05, lack of fit > 0.05). Moisture content has a very significant effect on WHC (*p* < 0.01) and chewiness (*p* < 0.01). Previous reports indicate that moisture content is the most critical factor affecting HMMA performance. Barrel temperature also has a significant effect on WHC (*p* < 0.01) and chewiness (*p* < 0.05). Additionally, interaction and quadratic terms did not show significant effects on the mentioned indicators (*p* > 0.05). Based on the importance of WHC over chewiness, the predicted optimal process parameters are moisture content 54.21%, screw speed 185.68 rpm, and barrel temperature 159.36 °C. Considering practical conditions, the optimal process variables for producing IMBP-based HMMAs in this experiment are a moisture content of 54%, screw speed of 186 rpm, and barrel temperature of 159 °C.

## 4. Discussion

This study systematically optimized extrusion parameters for mung bean protein-based high-moisture meat analogs (HMMAs), demonstrating that moisture content (54%) exerts the most significant influence on product properties, which aligns with Liu and Hsieh’s protein interaction theory regarding water-induced disruption of disulfide bonds. The observed water-holding capacity (251.66–370.47%) at elevated barrel temperatures (140–160 °C) extends Brishti et al.’s findings, highlighting mung bean protein’s superior thermal stability compared to hemp seed protein, while the optimal moisture range for oil absorption corroborates Hossain Brishti et al.’s viscosity threshold hypothesis, suggesting that mung bean protein requires less hydration than soy protein for optimal functionality. Notably, the IMBP-based HMMA exhibited higher integrity indices (62.67–96.47%) than pea protein analogs, consistent with Du et al.’s characterization of mung bean protein’s balanced hydrophilic-hydrophobic residues, and its nitrogen solubility index (26.60–46.13%) indicates better thermal stability than soy protein, making it suitable for high-temperature processing. The optimized extrusion conditions (54% moisture, 186 rpm, 159 °C) offer industrial advantages, including energy savings compared to soy protein extrusion and reduced equipment wear, while the achieved chewiness (2301–7089 g) matches commercial pea protein benchmarks, complemented by its allergen-free nature for broader market appeal. However, flavor masking remains a challenge, as noted by Brishti et al., necessitating future research into enzymatic modifications for umami enhancement, hybrid systems with pea protein for cost–performance optimization, and in vitro digestibility assessments to leverage mung bean’s high lysine content (6.2 g/100 g). These findings collectively advance the understanding of legume protein extrusion, bridging molecular insights with practical applications for sustainable meat alternatives.

## 5. Conclusions

The optimization of extrusion process parameters for producing high-moisture meat analogs (HMMAs) with 30% isolated mung bean protein was successfully achieved using Response Surface Methodology (RSM). Moisture content was identified as the most significant factor affecting water-holding capacity and chewiness, with increasing moisture leading to reduced fiber structure. Higher barrel temperatures were found to promote fiber structure formation. The optimal extrusion conditions were determined to be 54.21% moisture content, 185.68 rpm screw speed, and 159.36 °C barrel temperature, which were practically implemented as 54%, 186 rpm, and 159 °C, respectively.

Under these optimized conditions, the IMBP-based HMMAs demonstrated favorable water-holding capacity and appropriate chewiness, confirming the viability of isolated mung bean protein in meat analog applications. These findings suggest that further refinement of process parameters could enable the production of plant-based meat products with texture and functional properties more closely resembling animal meat, providing enhanced options for health-conscious consumers and sustainable food systems.

## Figures and Tables

**Table 1:** Central composite design for optimization of extrusion process variables.

X_1_	X_2_	X_3_
Moisture Content (%)	Screw Speed (rpm)	Barrel Temperature (°C)
50	150	150
50	200	140
50	200	160
50	250	150
55	150	140
55	150	160
55	200	150
55	200	150
55	200	150
55	250	140
55	250	160
60	150	150
60	200	140
60	200	160
60	250	150

**Table 2:** Physicochemical properties of isolated mung bean protein-based high-moisture meat analog according to process variables.

X_1_	X_2_	X_3_	Water Holding Capacity (%)	Water Absorption Capacity (%)	Oil Absorption Capacity (%)	Integrity Index (%)	Nitrogen Solubility Index (%)
M.C (%)	S.S (rpm)	B.T (°C)					
**50**	**150**	**150**	248.92 ± 19.53 ^d^	298.51 ± 3.14 ^h^	130.61 ± 5.12 ^h^	82.96 ± 1.46 ^d^	27.72 ± 0.95 ^gh^
**50**	**200**	**140**	251.66 ± 11.88 ^d^	312.52 ± 6.48 ^g^	156.63 ± 7.94 ^ef^	86.62 ± 0.14 ^bc^	26.60 ± 1.13 ^h^
**50**	**200**	**160**	282.26 ± 23.70 ^c^	319.97 ± 2.98 ^g^	161.87 ± 4.88 ^e^	79.66 ± 3.23 ^ef^	34.16 ± 1.58 ^cd^
**50**	**250**	**150**	255.03 ± 20.54 ^d^	316.65 ± 11.37 ^g^	138.91 ± 1.36 ^gh^	87.85 ± 0.81 ^bc^	31.19 ± 1.03 ^def^
**55**	**150**	**140**	255.00 ± 10.44 ^d^	336.78 ± 5.95 ^f^	174.27 ± 7.91 ^cd^	85.11 ± 2.47 ^cd^	30.03 ± 1.81 ^efg^
**55**	**150**	**160**	337.99 ± 5.97 ^b^	348.38 ± 3.53 ^e^	183.27 ± 4.18 ^abc^	63.17 ± 3.56 ^g^	46.13 ± 2.01 ^a^
**55**	**200**	**150**	282.21 ± 12.98 ^c^	346.43 ± 8.65 ^ef^	191.34 ± 4.32 ^ab^	88.12 ± 0.39 ^bc^	31.11 ± 1.91 ^def^
**55**	**200**	**150**	296.54 ± 4.97 ^c^	362.01 ± 1.78 ^d^	192.17 ± 6.42 ^ab^	88.76 ± 1.15 ^b^	29.45 ± 2.07 ^fgh^
**55**	**200**	**150**	283.84 ± 8.87 ^c^	359.50 ± 1.89 ^d^	186.13 ± 4.85 ^ab^	88.02 ± 0.11 ^bc^	29.69 ± 0.52 ^fgh^
**55**	**250**	**140**	246.85 ± 8.80 ^d^	352.50 ± 8.34 ^de^	164.67 ± 7.12 ^de^	96.47 ± 0.97 ^a^	28.61 ± 0.62 ^fgh^
**55**	**250**	**160**	287.12 ± 7.39 ^c^	359.51 ± 1.39 ^d^	193.56 ± 9.27 ^a^	78.86 ± 1.99 ^f^	33.07 ± 0.70 ^cde^
**60**	**150**	**150**	288.33 ± 9.00 ^c^	401.50 ± 4.30 ^b^	167.16 ± 9.82 ^de^	78.49 ± 1.65 ^f^	31.86 ± 1.63 ^def^
**60**	**200**	**140**	289.56 ± 10.68 ^c^	385.21 ± 5.38 ^c^	159.06 ± 5.11 ^e^	79.71 ± 1.26 ^ef^	35.69 ± 3.08 ^c^
**60**	**200**	**160**	370.47 ± 8.65 ^a^	425.66 ± 6.67 ^a^	147.98 ± 5.77 ^fg^	62.67 ± 2.56 ^g^	42.80 ± 2.77 ^b^
**60**	**250**	**150**	297.03 ± 23.80 ^c^	377.13 ± 7.03 ^c^	181.26 ± 5.22 ^bc^	82.52 ± 0.61 ^de^	36.00 ± 1.70 ^c^

X_1_—moisture content-M.C, X_2_—screw speed-S.S, X_3_—barrel temperature-B.T. Values with different letters in the same column indicate significant differences (*p* < 0.05) by Duncan’s multiple range test.

**Table 3:** Textural properties of isolated mung bean protein-based high-moisture meat analog according to process variables.

Run Order	X_1_	X_2_	X_3_	Texture Profile Analysis			Cutting Strength (g cm^−2^)	
	M.C (%)	S.S (rpm)	B.T (°C)	Springiness (%)	Cohesiveness (%)	Chewiness (g)	Vertical Direction	Parallel Direction
**1**	**50**	**150**	**150**	94.62 ± 0.53 ^cde^	85.63 ± 0.69 ^abc^	6286.45 ± 213.80 ^b^	1318.25 ± 120.46 ^bc^	1026.07 ± 160.41 ^a^
**2**	**50**	**200**	**140**	93.78 ± 1.14 ^ef^	86.27 ± 2.45 ^ab^	5625.87 ± 212.10 ^c^	1164.16 ± 81.50 ^d^	797.62 ± 33.81 ^d^
**3**	**50**	**200**	**160**	92.57 ± 0.55 ^g^	87.11 ± 0.91 ^a^	5415.51 ± 378.55 ^cd^	1595.01 ± 102.28 ^a^	1027.58 ± 73.89 ^a^
**4**	**50**	**250**	**150**	94.73 ± 1.15 ^bcde^	86.33 ± 0.76 ^ab^	7089.33 ± 168.53 ^a^	1348.36 ± 67.40 ^b^	912.56 ± 56.78 ^bc^
**5**	**55**	**150**	**140**	95.26 ± 1.13 ^abcd^	85.33 ± 0.97 ^abc^	5321.18 ± 209.94 ^d^	996.66 ± 55.12 ^fg^	705.42 ± 21.31 ^de^
**6**	**55**	**150**	**160**	92.73 ± 1.16 ^fg^	85.72 ± 0.78 ^abc^	3818.07 ± 272.59 ^f^	1206.44 ± 164.38 ^cd^	994.04 ± 76.62 ^abc^
**7**	**55**	**200**	**150**	95.40 ± 1.35 ^abc^	84.49 ± 3.25 ^abc^	5315.10 ± 287.43 ^d^	1137.43 ± 115.36 ^de^	896.58 ± 60.60 ^c^
**8**	**55**	**200**	**150**	95.26 ± 0.77 ^abcd^	85.20 ± 1.44 ^abc^	5986.49 ± 184.81 ^b^	1198.23 ± 46.79 ^cd^	1007.57 ± 64.20 ^ab^
**9**	**55**	**200**	**150**	96.41 ± 0.78 ^a^	84.38 ± 3.73 ^abc^	5646.54 ± 166.51 ^c^	1272.55 ± 99.09 ^bcd^	911.55 ± 79.80 ^bc^
**10**	**55**	**250**	**140**	95.99 ± 0.82 ^ab^	85.50 ± 1.11 ^abc^	5993.87 ± 400.22 ^b^	928.05 ± 117.87 ^fg^	645.00 ± 84.31 ^e^
**11**	**55**	**250**	**160**	95.44 ± 0.73 ^abc^	86.08 ± 0.95 ^abc^	4206.93 ± 204.52 ^e^	1190.59 ± 67.34 ^cd^	1020.59 ± 92.47 ^a^
**12**	**60**	**150**	**150**	94.03 ± 0.72 ^de^	83.79 ± 0.96 ^bc^	2703.68 ± 62.74 ^h^	945.09 ± 148.90 ^fg^	923.99 ± 61.13 ^abc^
**13**	**60**	**200**	**140**	96.07 ± 1.07 ^a^	83.37 ± 4.68 ^c^	2796.72 ± 358.25 ^h^	755.79 ± 176.35 ^h^	678.92 ± 75.20 ^e^
**14**	**60**	**200**	**160**	96.32 ± 0.85 ^a^	85.80 ± 1.21 ^abc^	2301.10 ± 166.41 ^i^	1030.82 ± 76.18 ^ef^	675.79 ± 129.54 ^e^
**15**	**60**	**250**	**150**	95.76 ± 1.32 ^abc^	86.13 ± 1.37 ^abc^	3250.06 ± 192.44 ^g^	867.42 ± 71.90 ^gh^	780.04 ± 43.84 ^d^

X_1_—moisture content—M.C, X_2_—screw speed—S.S, X_3_—barrel temperature—B.T. Values with different letters in the same column indicate significant differences (*p* < 0.05) by Duncan’s multiple range test.

**Table 4:** Analysis of variance of regression models for the water holding capacity and chewiness of isolated mung bean protein-based high-moisture meat analogs.

Source	Water Holding Capacity		*p* Value	Chewiness
	*p* Value	Significant		Significant
**Model**	0.0179	*	0.0065	**
**X_1_-moisture content**	0.0041	**	0.0003	**
**X_2_-screw speed**	0.3344		0.1581	
**X_3_- barrel temperature**	0.0024	**	0.0375	*
**X_1_ X_2_**	0.9329		0.8129	
**X_1_ X_3_**	0.1463		0.7297	
**X_2_X_3_**	0.2043		0.7935	
**X_1_ ^2^**	0.9229		0.0308	*
**X_2_^2^**	0.0901		0.9442	
**X_3_^2^**	0.2387		0.0311	*
**Lack of Fit**	0.1674		0.2447	
**R^2^**	0.9335		0.9569	
**Adj R^2^**	0.8139		0.8792	
**C.V. %**	5.14		10.73	

*—Significant at 0.05 level, **—Significant at 0.01 level.

## Data Availability

The original contributions presented in this study are included in the article. Further inquiries can be directed to the corresponding author.
